# Minimizing the impact of microorganism intrusion on the concrete physical and mechanical properties with nickel waste as a partial substitution for cement

**DOI:** 10.1016/j.heliyon.2023.e13303

**Published:** 2023-01-31

**Authors:** Hanafi Ashad

**Affiliations:** a Department of Civil Engineering, Universitas Muslim Indonesia, Makassar, South Sulawesi, 90232, Indonesia

**Keywords:** Intrusion, Microorganism, Minimizing impact, Nickel slag

## Abstract

This study examined the impact of microorganism intrusion on concrete's physical and mechanical properties and efforts to minimize the effect by using nickel waste as a partial substitute for cement. The microorganisms resulted from the natural fermentation of coconut water which intruded into the concrete material, harming the concrete's physical and mechanical properties. Physical and mechanical properties observed were porosity, permeability, and compressive strength. The results indicated that the intrusion of microorganisms into the concrete material increased porosity and permeability and decreased the compressive strength of the concrete. Using nickel slag as a partial cement substitution material with an optimal percentage of 15% was employed to overcome these impacts.

## 1 Introduction

Along with the rapid development of infrastructure, the use of concrete as a structural component material increases in the construction of buildings, roads, docks, and other infrastructure. It becomes one of the best choices because it is durable, malleable, and able to be fabricated with good physical and mechanical properties, such as low porosity and high compressive strength.

Damage to concrete structural components is not only caused by excessive loading but also due to the influence of the surrounding environment [1]. Damage to structural components due to microorganism attacks is often found in Indonesia, especially in environments where these structural components are in direct contact with the environment where microorganisms grow and develop, for example, in various traditional markets. The Ministry of Trade of the Republic of Indonesia reports that the number of traditional markets in 2021 will be 16,175 units spread throughout Indonesia with an investment value of trillions of rupiah. This incident must have a solution because, in addition to having high investment value, it also maintains the safety of infrastructure users.

Various ways to overcome the damage of concrete's structural components include; using water-resistant and high-quality concrete by using new material innovations. In the last few decades, new materials in concrete construction have been used, such as industrial waste like fly ash, silica fume, blast furnace slag, and others [3, 4, 5].

Nickle waste, commonly called nickel slag, is one of the industrial wastes used in concrete construction. The waste is interesting to study because the deposit is quite large, and the process is through smelting nickel ore with a temperature of 1550 °C [6, 7]. Materials obtained from combustion at high temperatures generally contain high silicate oxide (SiO_2_) or abbreviated S chemical compounds [8, 9].

The use of nickel slag as a supplement material to make concrete mixtures has several advantages, including; improving concrete's performance and reducing cement production costs and CO_2_ gas emissions [10, 11]. In addition, using nickel slag as a construction material is one of the solutions to reducing environmental pollution [12].

Two of the four main chemical compounds of cement, namely tricalcium silicate (3CaO·SiO_2_ or abbreviated C_3_S) and dicalcium silicate (2CaO·SiO_2_ or abbreviated C_2_S), when chemically reacted with water (H_2_O or abbreviated as H), will produce two new chemical compounds, namely calcium silicate hydrate (CaO·SiO_2_·H_2_O or abbreviated CSH) and calcium hydroxide (Ca(OH)_2_ or abbreviated as CH) as shown in equations (1), (2). Calcium silicate hydrate as the main product is solid, while calcium hydroxide as a by-product is soluble [13, 14].

The chemical compound calcium hydroxide (CH) in equations (1), (2) in porous concrete is very susceptible to attack by microorganisms because the compound is free lime which is soluble and can be a nutrient for microorganisms [15].

2(3CaO·SiO_2_) + 6H_2_O → 3CaO·2SiO_2_·3H_2_O + 3Ca(OH)_2_

or

(1) 2C_3_S + 6H → C_3_S_2_H_3_ + 3CH

2(2CaO·SiO_2_) + 4H_2_O → 3CaO·2SiO_2_·3H_2_O + Ca(OH)_2_

or

(2) 2C_2_S + 4H → C_3_S_2_H_3_ + CH

The innovation of new materials from nickel waste containing the chemical compound silica oxide (S) has the potential to chemically modify calcium hydroxide (CH) into a secondary hydrated calcium silicate compound (CSH), shown in equation (3). The result of this chemical modification is also known as a pozzolanic reaction [16].

(3) CH + S → CSH_(sekunder)._

Research on efforts to improve the properties of concrete by using mineral admixtures has been going on for many years. Recently, the mineral admixtures used in concrete production have significantly increased because it is more environmentally friendly. Some modern cement increase their initial strengths with more formation of Ca(OH)_2_, but this can negatively affect the durability and cost of concrete [3].

Research reports on the use of nickel waste as a substitute for cement are still lacking, so the authors can only add limited up-to-date information.

Bunga Yubi Nabiila et al. (2019) suggested that nickel waste powder can be used as an alternative material for cement supplements which is environmentally friendly.

Based on the description above, this research examines the optimal use of nickel waste as a partial cement substitution material to minimize the negative impact of microorganism intrusion on concrete's physical and mechanical properties.

Furthermore, the significance of this research is that it is at least a solution to overcoming the impact of the exploitation of natural resources and the environment, as well as an alternative solution for the proper functioning of buildings under conditions of intrusive microorganisms.

## 2 Experimental program

### 2.1 Materials

The materials used include; Portland Cement, natural sand, crushed stones, nickel slag, and water as a solvent, with the following specifications.

- Portland Cement; Production of PT. Semen Tonasa type I
- Natural sand; obtained from Bili-Bili, Gowa Regency, South Sulawesi
- Crushed Stone: obtained from Bili-Bili, Gowa Regency, South Sulawesi
- Water; obtained from PDAM Makassar City, South Sulawesi
- Nickel Slag; Production of PT. Vale, Soroako, South Sulawesi

Nickel slag-in the form of lumps-was ground to a powder that was finer granules than cement granules. The milled powder used was determined with a specific surface value greater than or equal to 320 m^2^/kg. To test the number of colony microorganisms in concrete, partial replacement of cement with nickel slag powder was 0%, 10%, 12%, 14%, 16%, 18%, and 20%. These proportions aimed to determine the optimum nickel slag percentage used in the next test.

The chemical composition of cement and nickel slag is shown in Table 1.

**Table 1:** Chemical composition of cement and nickel slag.

Chemical compound	Unit	Cement	Nickel slag
SiO_2_	%	21,83	40,83
CaO	%	64,17	5,95
Al_2_O_3_	%	6,65	3,86
Fe_2_O_3_	%	3,22	23,44
MgO	%	1,08	14,07
Na_2_O	%	–	1,92
K_2_O	%	–	0,08
NiO	%	–	3,76
SO_3_	%	1,84	0,23
S	%	–	0,09
LoI	%	0,81	5,02

Microorganisms are obtained through naturally fermented coconut water media.

### 2.2 Sample preparation

Cylindrical concrete specimens with a diameter of 150 mm and a height of 300 mm are used for various tests, including; testing the number of microorganism colonies in concrete at various depths, porosity testing, and compressive strength. For permeability testing, using a cube-shaped specimen measuring 150 × 150 × 150 mm. Concrete specimens were made with a water-binder ratio (w/b): 0.30, 0.45, and 0.55, respectively.

Concrete specimens were prepared with the mixed composition as shown in Table 2. The specimens from each mixture were given two kinds of treatment, namely the treatment of being immersed in water (normal) and the treatment of being immersed in a solution containing microorganisms (intrusion of microorganisms) for 210 days.

**Table 2:** Composition of concrete mixture in 1 m^3^

w/b	NS		C	W	FA	CA	SP
	%	kg	(kg)	(kg)	(kg)	(kg)	(cc)
0,55	0	0	365	203	770	925	4270
	10	37	328				3843
	12	44	321				3758
	14	51	314				3673
	16	58	307				3587
	18	66	299				3502
	20	73	292				3416
0,45	0	0	498	226	714	957	6130
	10	50	448				5513
	12	60	438				5390
	14	70	428				5268
	16	80	418				5145
	18	90	408				5023
	20	100	398				4900
0,30	0	0	576	184	668	1090	7044
	10	58	518				6324
	12	69	507				6184
	14	81	495				6043
	16	92	484				5903
	18	104	472				5762
	20	115	461				5622

Where: C: cement. NS: nickel slag. W: water. b: binder. FA: fine aggregate. CA: coarse aggregate. SP: superplasticizer.

The 210 days of treatment was determined considering that this time was sufficient to provide information to predict the long-term condition of concrete properties.

### 2.3 Testing procedure

The first step is identifying the type of microorganism that grows on coconut water fermentation media.

The second step was to test the number of microorganism colonies in the concrete with the percentage of nickel slag as mentioned above.

After knowing the optimum percentage of nickel slag, further testing was the quantity of calcium hydroxide (Ca(OH)_2_) and the physical and mechanical properties of concrete; porosity, permeability, and compressive strength.

The composition of the concrete mixture for each specimen is shown in Table 2.

The porosity test used the ASTM C642-90 standard. The permeability coefficient test used the DIN-1045 test standard. Meanwhile, the concrete compression test used the ASTM C136-06 test standard. The tests were carried out at the age of the concrete and/or the intrusion time from 28 days to 210 days.

**Fig. 1:**
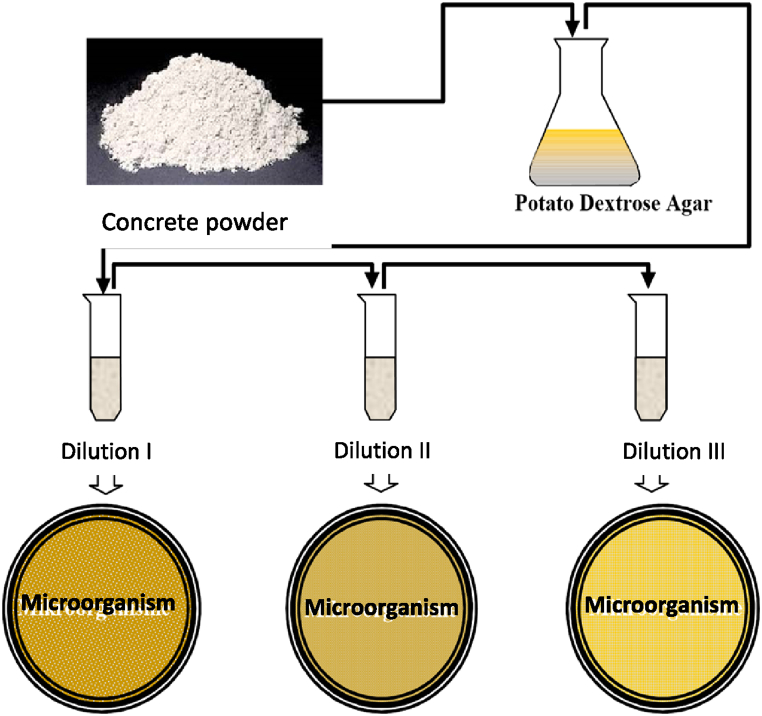
Testing microorganism procedure on concrete sample.

The presence of microorganisms in the concrete specimen was identified by drilling at three points, each with a depth of *D* = 0–25 mm, *D* = 25–50 mm, and *D* = 50–75 mm. The procedure for testing microorganisms in the concrete is in Fig. 1. The calculation of microorganism colonies used the Plate Count Standard (PCS) with the following formula:

(4) $$Cm=\frac{number\;of\;colonies\;x\;dilution\;factor}{volume\;of\;sample\;plated}\;\left(cfu/ml\right)$$

After the data on the number of microorganism colonies was calculated according to equation (4), then a graph of the relationship between the number of microorganisms colony and the percentage of nickel slag was made, making it easier to obtain the optimum percentage use of nickel slag.

Based on the optimum percentage, concrete specimens were then made for tests, including; quantity of calcium hydroxide (Ca(OH)_2_), porosity, coefficient of permeability, and compressive strength. The test results were presented in three relationship graphs; calcium hydroxide versus time, porosity versus time, and coefficient of permeability versus time.

### 2.4 Nomenclature of sample

The nomenclature of the test object is as follows:

- NI-0; Concrete non-intruded by microorganism without nickel slag.
- NI–S; Concrete non-intruded by microorganism with the optimum percentage of nickel slag.
- I-0; Concrete intruded by microorganism without nickel slag.
- I–S; Concrete intruded by microorganism with the optimum percentage of nickel slag.

## 3 Result and discussion

### 3.1 Identification of microorganisms

As previously stated, microorganisms are obtained through a natural fermentation process from coconut water media. The results of the identification of the types of microorganisms that grow through this process are the dominant groups of *Aspergillus niger* and *Sacchromycodes niger*. Both of these groups have the characteristic of colonial growth with a diameter between 0.05 μm and 0.5 μm.

Furthermore, through *chromatographic* testing with the High-Performance Liquid Chromotography method, the two groups of microorganisms, in their metabolic processes produce acetic acid (CH_3_COOH).

### 3.2 Profile of microorganisms in concrete

**Fig. 2:**
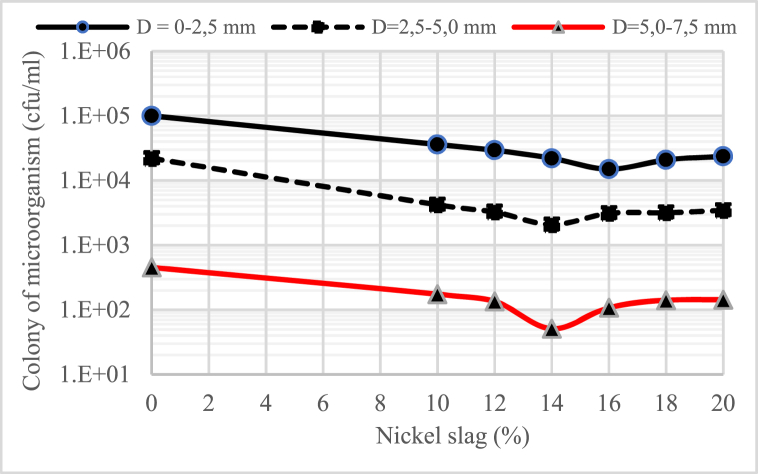
Profile of colony microorganism on concrete for water-binder ratio (w/b) = 055.

**Fig. 3:**
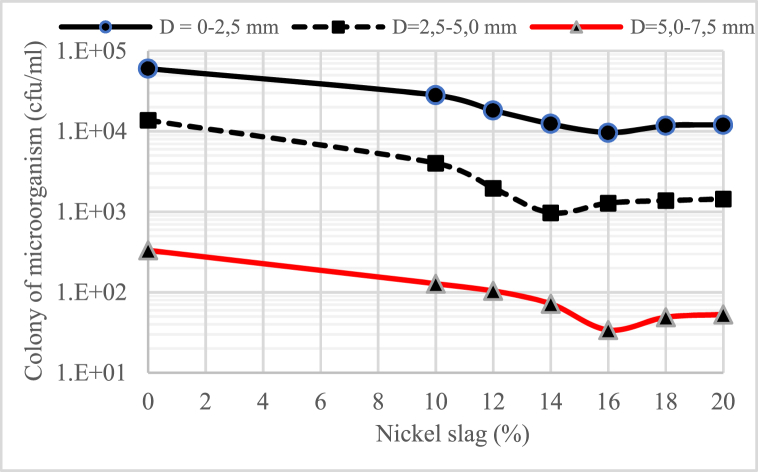
Profile of colony microorganism on concrete for water-binder ratio (w/b) = 045.

**Fig. 4:**
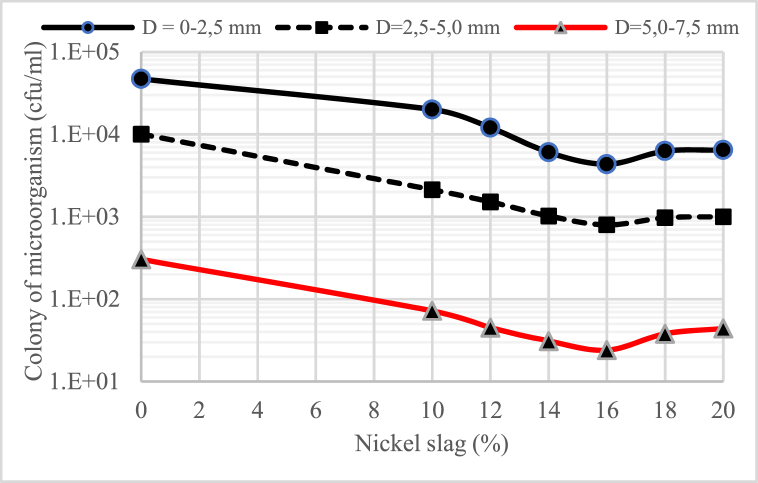
Profile of colony microorganism on concrete for water-binder ratio (w/b) = 0,30.

Fig. 2, Fig. 3, Fig. 4 show the profile of microorganisms in the concrete material, where the water-binder ratio (w/b) is also very influential in the number of microorganism colonies that can be intruded into the concrete. It can be said that the lower the water-binder ratio (w/b), the less the number of microorganism colonies, especially in the concrete that uses nickel slag. The number of microorganisms intruded into the concrete decreased with the increasing depth of the sampling point (D).

It was also found that the number of microorganisms in the concrete material decreased significantly with the increase in the portion of nickel slag as a partial cement substitute. The graphs shown in Fig. 2, Fig. 3, Fig. 4 generally indicate that the portion of nickel slag that can withstand the rate of intrusion of microorganisms was between 14% and 16%. This means that in the nickel slag portion range, the pozzolanic reaction between silica oxide (SiO_2_) and calcium hydroxide (Ca(OH)_2_) can make the concrete more solid due to the formation of a new chemical compound, namely secondary calcium silicate hydrate (CSH) [13].

The change in chemical compounds of calcium hydroxide (Ca(OH)_2_) into solid secondary calcium silicate hydrate (CSH) was the effect of nickel slag, known as the pozzolanic effect [13].

In the biological aspect, this change has a negative effect on the survival of microorganisms because of the loss of nutrients for the life of microorganisms that come from calcium hydroxide (Ca(OH)_2_) in the concrete material.

Considering the microorganism profile mentioned above, concrete's physical and mechanical properties were tested using a 15% nickel slag portion.

### 3.3 Calcium hydroxide compound change

In this study, observations of changes were only reported in the concrete with a water-cement ratio (water-binder ratio (w/b) = 0.55). Likewise, the portion of nickel slag usage was limited to 15% as it was the optimal percentage, as shown in Fig. 2, Fig. 3, Fig. 4 and the results of previous research.

**Fig. 5:**
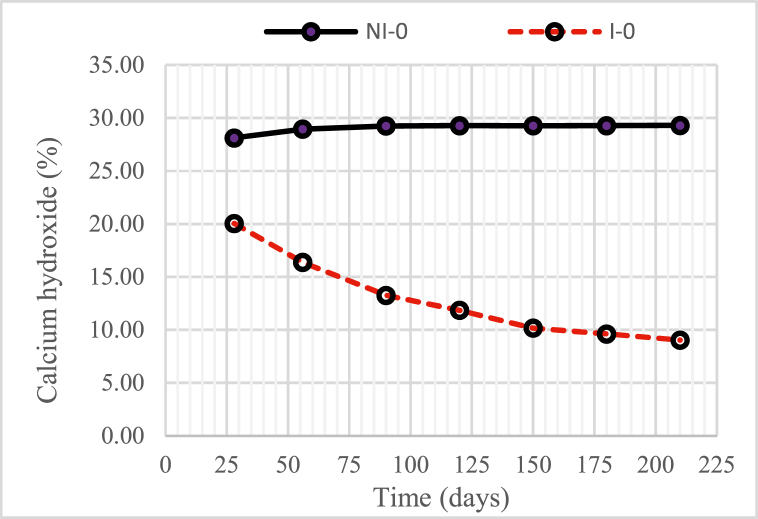
Changes in the quantity of calcium hydroxide (Ca(OH)_2_) a compound in concrete without nickel slag or with w/b = 0.55.

**Fig. 6:**
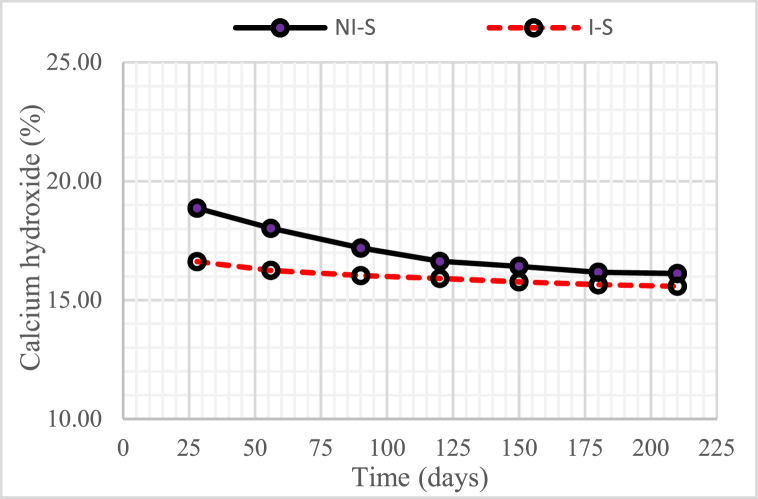
Changes in the quantity of calcium hydroxide (Ca(OH)_2_) a compound in concrete with nickel slag or with w/b = 0.55.

Changes in Ca(OH)_2_ compounds as a function of time, both intruded and non-intruded with microorganisms, were shown in Fig. 5 (for concrete without nickel slag) and Fig. 6 (for concrete with 15% nickel slag).

Fig. 5 shows two curves with different patterns of change in the chemical compound Ca(OH)_2_, the first in the non-intruded microorganism concrete (without nickel slag) increasing until the concrete age reaches 90 days and then tended to be constant up to 210 days. Second, the intruded microorganism concrete (without nickel slag) reduced significantly over time. This increase occurred as an indication that the process of formation of Ca(OH)_2_ compounds as by-products of the chemical reaction of tricalcium silicate (C_3_S) and dicalcium silicate (C_2_S) from cement and water only lasted until 90 days. On the other hand, the reduction occurs because the colony of microorganisms makes Ca(OH)_2,_ a nutrient for survival in the concrete.

Fig. 6 shows that the compound Ca(OH)_2_ in non-intruded concrete by microorganisms (with 15% nickel slag) was significantly reduced until the concrete age reached 120 days. It occurred because of the further chemical reaction between the Ca(OH)_2_ by-product of the chemical reaction of cement and water with silica oxide (SiO_2_) from nickel slag [3]. The chemical reaction produced a secondary solid Calcium Silicate Hydrate (CSH) compound. Furthermore, Ca(OH)_2_ in microorganism-intruded concrete (with 15% nickel slag) decreased slightly from time to time and even tended to be constant, particularly from 120 days to 210 days.

### 3.4 Porosity

**Fig. 7:**
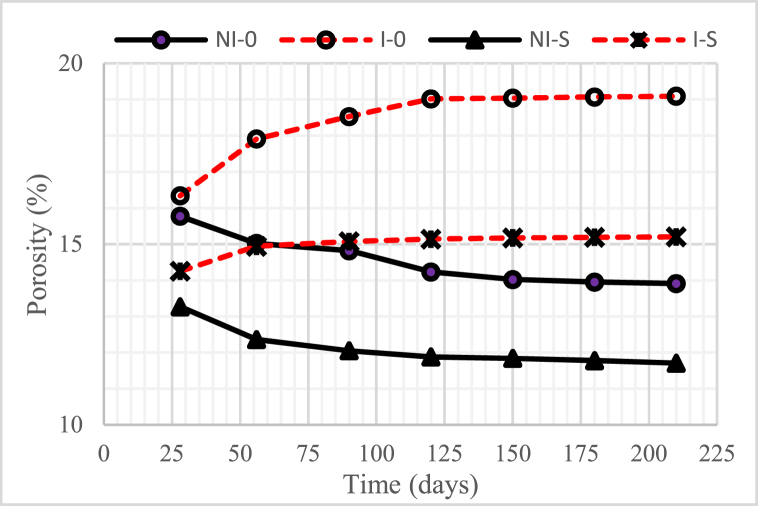
Concrete porosity for w/b = 0.55.

**Fig. 8:**
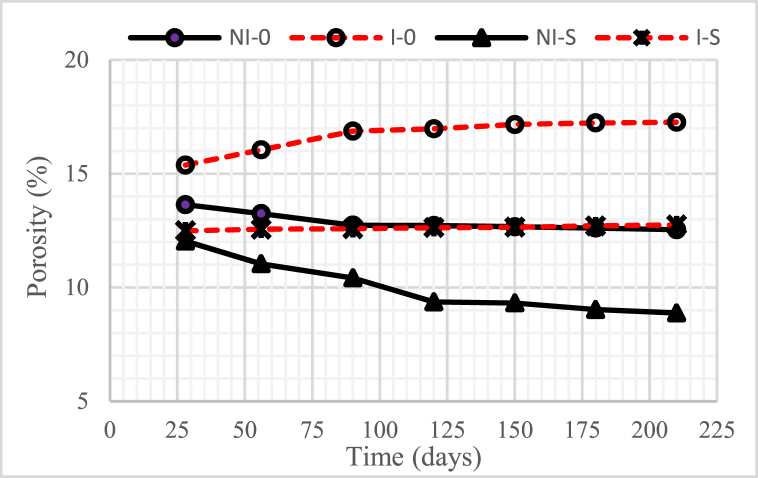
Concrete porosity for w/b = 0.45.

**Fig. 9:**
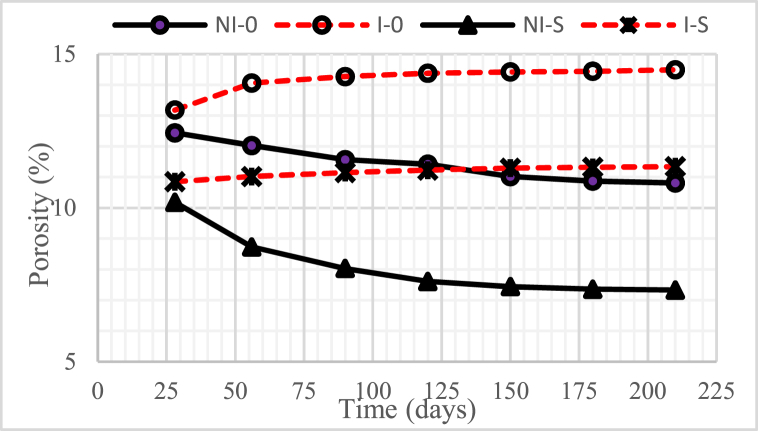
Concrete porosity for w/b = 0.30.

Fig. 7, Fig. 8, Fig. 9 illustrate the porosity of concrete with a water-binder ratio (w/b) of 0.55, 0.45, and 0.30, respectively. The graphs indicate that the higher the water-binder ratio (w/b), the higher the porosity of the concrete.

The non-intruded microorganism concrete, with or without nickel slag, after 28 days experienced a decrease in porosity, except when the concrete age was from 150 days to 210 days, which tended to be constant. A significant drop in porosity occurred in concrete with 15% nickel slag. This decrease was due to the effect of pozzolanic nickel slag as a partial substitution for cement. In addition, it was also due to the physical impact of nickel slag powder, where the specific surface value was more significant than the cement-specific surface [6,7,17]. The mentioned pozzolanic effect was to change the chemical compound Ca(OH)_2_ into secondary Calcium Silicate Hydrate (CSH) through a further chemical reaction with silica oxide (SiO_2_) from nickel slag. This follow-up reaction is known as the Pozzolanic Reaction [13].

On the other hand, for concrete intruded by microorganisms, both with and without nickel slag, after 28 days, the porosity increased except for the intrusion duration of 150 days–210 days, which tended to be constant. A very significant increase in porosity occurred in the concrete without nickel slag. This increase occurred due to the formation of voids because less Ca(OH)_2_ was consumed by colony microorganisms.

Apart from less Ca(OH)_2_, pozzolanic effects, and physical effects, the porosity was also determined by the water-cement ratio. The higher the water-binder ratio (w/b), the more potential it was to cause many interconnected pores. These pores generally contain interconnected air or water called concrete capillaries [18]. The concrete capillaries will still exist even though the water evaporates, resulting in a reduction in the density of the concrete. With increasing pore volume, the porosity of the concrete would also increase, which could reduce the strength of the concrete [19].

Based on the preceding, it can be concluded that using nickel slag as a partial substitution of cement with a portion of 15%, both in the intruded and non-intruded conditions of microorganisms, can significantly reduce the porosity of the concrete.

### 3.5 Coefficient of permeability

**Fig. 10:**
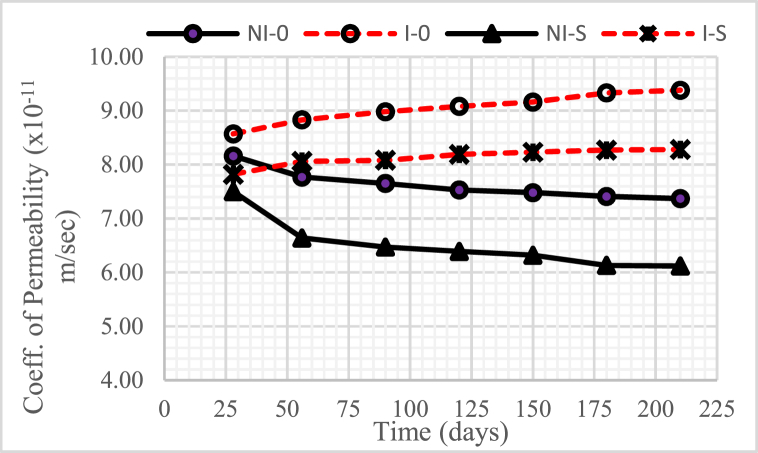
The coefficient of permeability of concrete for w/b = 0.55.

**Fig. 11:**
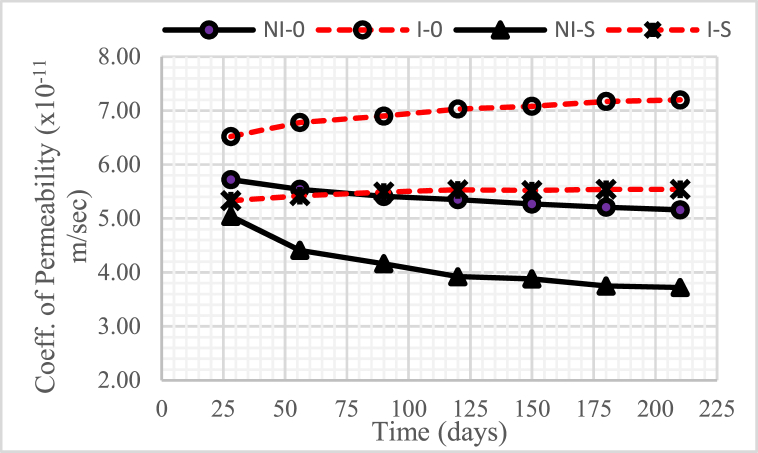
The coefficient of permeability of concrete for w/b = 0.45.

**Fig. 12:**
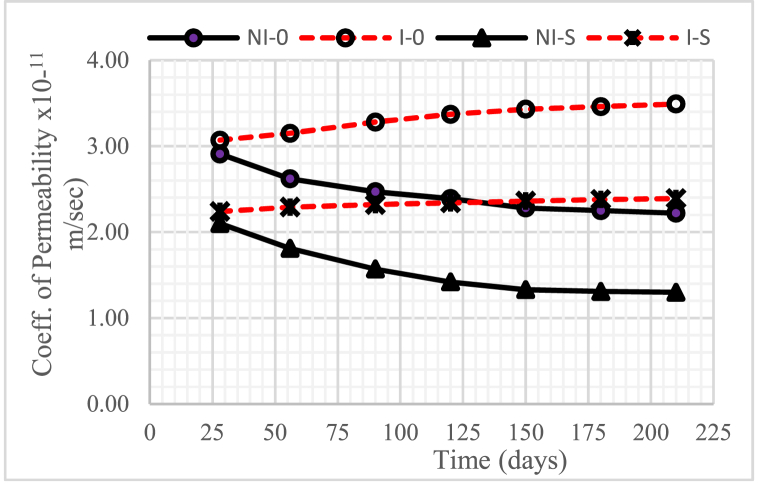
The coefficient of permeability of concrete for w/b = 0.30.

Fig. 10, Fig. 11, Fig. 12 show the changes in the permeability coefficient of concrete with a water-binder ratio (w/b) of 0.55, 0.45, and 0.30, respectively. The graphs shown in the three figures show that the higher the water-binder ratio (w/b), the higher the permeability coefficient.

In general, concrete intruded by microorganisms, both with and without nickel slag, experienced an increase in the permeability coefficient from time to time, except when the intrusion duration was 150 days–210 days, which tended to be constant. This increase occurred because of the reduced Ca(OH)_2_ due to consumption by colony microorganisms so that it left the pores.

Meanwhile, the permeability coefficient decreased from time to time in unintruded concrete by microorganisms, both without and with nickel slag, except when the concrete was 150 days–210 days old. This decrease occurred from the pozzolanic effect of nickel slag or the physical effect of the nickel slag powder itself. The pozzolanic result mentioned is a further chemical reaction between Ca(OH)_2_ and silica oxide (SiO_2_) nickel slag, which is commonly called the Pozzolanic Reaction [13]. Meanwhile, the physical effect was obtained through nickel slag with a specific surface value greater than the particular surface value of cement [2].

Therefore, using nickel slag with a portion of 15% can significantly reduce the permeability coefficient of concrete, both under normal conditions and intruded by microorganisms.

Apart from the effect of reduced Ca(OH)2, pozzolanic effect, and physical effect, the permeability coefficient was also highly determined by the water-cement ratio. The higher the water-binder ratio (w/b), the more potential it is to cause many interconnected pores so that the permeability of the concrete becomes high [20].

### 3.6 Compressive strength

**Fig. 13:**
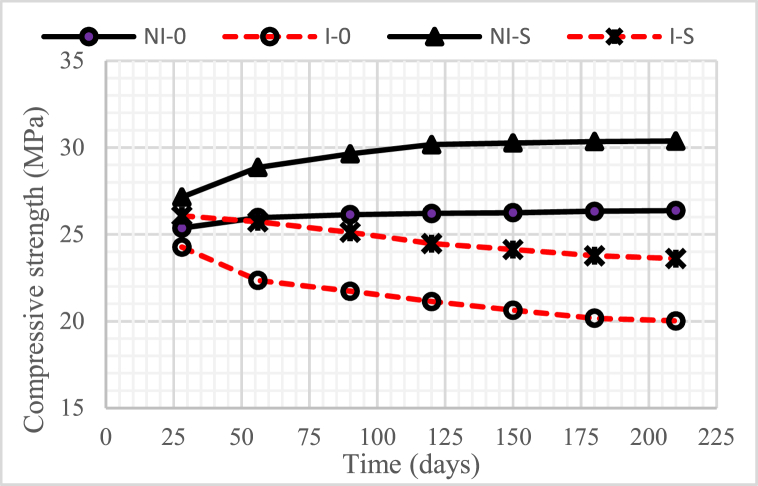
Compressive strength of concrete for w/b = 0.55.

**Fig. 14:**
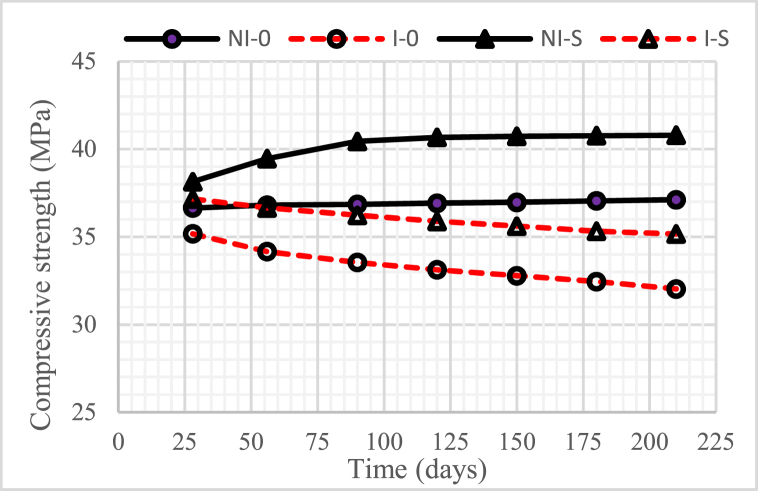
Compressive strength of concrete for w/b = 0.45.

**Fig. 15:**
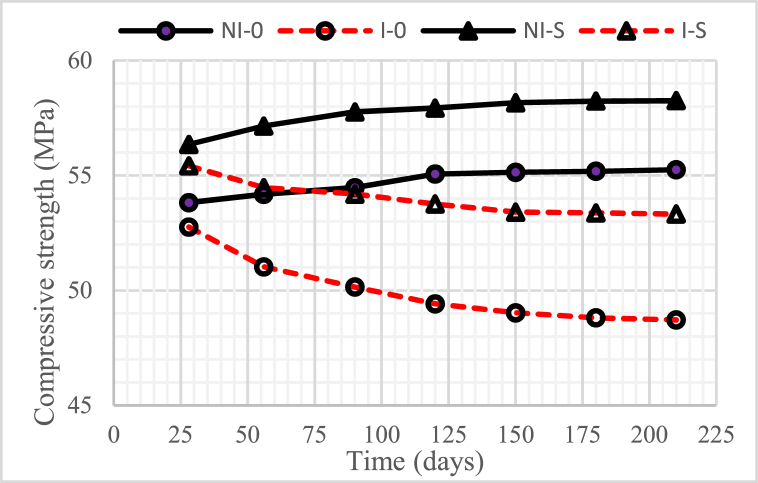
Compressive strength of concrete for w/b = 0.30.

Fig. 13, Fig. 14, Fig. 15 show the development of the compressive strength of concrete, both in the intruded and non-intruded by microorganisms, with a water-binder ratio (w/b) = 0.55, 0.45, and 0, 30. As with the physical properties discussed previously, the compressive strength of concrete increased as the water-binder ratio (w/b) decreased.

The graphs in the three figures indicate that the compressive strength of unintruded concrete by microorganisms increased with the age of the concrete. A very significant increase was obtained in concrete with 15% nickel slag. This increase occurred until the age of the concrete reached 120 days, after which it tended to be constant.

The compressive strength of concrete intruded by microorganisms decreased with increasing duration of intrusion. A very significant reduction occurred in the concrete without nickel slag. Especially for concrete with 15% nickel slag, the decrease in compressive strength was not too high compared to concrete without nickel slag, where the time for the decline only lasted until the intrusion time of 180 days and, after that, tended to be constant.

The increase in compressive strength, especially in concrete using nickel slag, was a positive contribution from the pozzolanic reaction that produced a solid compound called secondary Calcium Silicate Hydrate (CSH) [6]. Therefore, the compressive strength of concrete was highly correlated with CSH, where the compressive strength of concrete increased along with CSH increase.

The decrease in the compressive strength of concrete due to the intrusion of microorganisms was caused by the voids formed due to the reduction of Ca(OH)_2_ because microorganisms consumed it.

## 4 Conclusion

Based on the results of the analysis and discussion, it can be concluded as follows:

1. The intrusion of microorganisms into the concrete material has a significant and negative impact on the physical and mechanical properties of the concrete.
2. The use of nickel slag as a partial substitute for cement in concrete with a low water-cement ratio was found to minimize the number of colonies of microorganisms intruding into the concrete material.
3. The uses of nickel slag as a partial substitute for cement can reduce the negative impact of microorganism intrusion on the physical and mechanical properties of the concrete.
4. The optimum percentage of nickel slag that can minimize this impact is 15%.

To increase the replicability and reproducibility of the results of this study, it is suggested to carry out further research on durability and application in reinforced concrete structural components.

## Author contribution statement

Hanafi Ashad, Dr.: Conceived and designed the experiments; Performed the experiments; Analyzed and interpreted the data; Contributed reagents, materials, analysis tools or data; Wrote the paper.

## Funding statement

Mr. Hanafi Ashad was supported by PT. Semen Tonasa [0173/ST-SIG/K1/VII/2021].

## Data availability statement

Data included in article/supp. material/referenced in article.

## Declaration of interest's statement

The authors declare no competing interests.
